# Cheap Prescribing

**Published:** 1916-07-29

**Authors:** 


### The Hospital

The Workers' Newspaper of Administrative Medicine and Institutional Life, Administration, National Insurance and Health.

No. 1573, Vol. LX. SATURDAY, JULY 29, 1916. PRICE ONE PENNY.

### CHEAP PRESCRIBING.

Questions not only of pounds and shillings but also of pence have taken on a special note of urgency in the present stress of public affairs. While this is true generally, the proposition applies with particular force to the finance of the National Insurance Act. Expert investigations show the need for anxiety in relation to the whole field, and very properly, therefore, consideration is being given to the possibility of improving the situation. With the question in its wider aspects we are not immediately concerned. Our concern is solely with the attempt to reduce expenses by cheapening the cost of medicines. It is well known that a considerable number of individual practitioners have been in trouble with the Insurance Committees because their prescriptions have shown an average cost in excess of the sum contemplated by the Act and in excess also of the charge incurred by their fellow-practitioners. That in some of these cases there has been carelessness or thoughtless extravagance can hardly be doubted, and the practitioners in question have had to pay for their idiosyncrasies by a surcharge which has sometimes amounted to a substantial sum. In other instances the justice of the fine may be questioned, but we do not propose to challenge any one or other of the individual verdicts. It is quite right that the spending of public money—for this is what is involved—should be subject to some form of supervision, and that those who are charged with its distribution should be reminded, imperatively if necessary, of their responsibilities. Yet in medical practice the first word is not economy but efficiency, and the process of reducing expenses must be conducted in accordance with this maxim. Certainly it is hardly an atmosphere favourable to the successful treatment of disease when the practitioner has to keep prominently before his mind the necessity of constructing cheap prescriptions, lest perchance he should be compelled to pay for the medicines out of his own pocket. This, however, is the position which has been in some measure established by the practice of surcharging the practitioner as above described, and however necessary it may be on grounds of economy, it is not a happy development from the professional point of view. One of the reasons advanced for taking away from the practitioner working under the Insurance Act the right of supplying medicines to his patients was that he would be removed from the temptation of prescribing anything less than the best. But the practical development of affairs has pushed to the front the very issue which the new regulation was believed to have closed, and the cheapness of his prescriptions has necessarily become an anxious thought to the panel practitioner. He must, whate'er betide, keep his average within the standard, or he will hardly avoid a summons from his Insurance Committee and the risk of a more or less substantial reduction from his fees. In the existing state of affairs we should agree that the present practice cannot be avoided, but the position is not a pleasant one. Perhaps it may be quoted as an example of what happens to medicine when it is tied in official bonds, and it may serve to discourage those who think that such bonds might be multiplied with advantage.

While the fear of the surcharge doubtless helps to economy, a more positive aid to the same end is the "stock mixture." As this can be prepared in bulk and made in advance, it saves time in dispensing, and so the cost of the individual prescription is reduced, and it further tempts the prescriber by the ease and simplicity with which it can be ordered. An important consideration is the ability of the mixture to be stored without deterioration of its therapeutic values, and in individual cases this has been satisfactorily established and the use of some of these mixtures has received the sanction of certain Insurance authorities. Here, again, what is desirable as an ideal has to yield to the pressure of practical affairs. Strictly speaking, the practitioner ought to fit his prescription to the needs of his patient, and not, on the contrary, to attach the patient to a prescription ready made. Yet it must be admitted that the "stock mixture" has a considerable vogue, and for the sake of economy in both time and money the hospitals, and more particularly the out-patient departments, cultivate it largely. Thus it happens that the student in his earliest days is encouraged in the practice, and the habit is apt to hold him in later years. It is difficult to deny that one of the unfortunate consequences of such a method is the ordering of a considerable amount of unnecessary medicine. The patient repeats his or her woes, real or imaginary, and the doctor, as the easiest way out of an impossible situation, repeats the prescription. Of course, it would be well, if other and more helpful measures were available, to turn the patient in the appropriate direction and to discontinue the unnecessary medicine. But suitable dietetic measures or climatic influences, or such methods as massage and the rest, are often out of reach on account of the expense involved, and all that remains is to make the best of a situation which cannot be fully remedied. According to the patient—who on such an issue ought to be an authority—the bottle of medicine does good, and hence mere kindliness prompts its repetition. Even for patients whose circumstances permit a wider choice experience often shows a ready willingness to take medicine with a decided unwillingness to take good advice. Of course, the doctor ought to cultivate his educational opportunities and to impress on his patients the need of doing other things than the swallowing of physic. But many members of his congregation are held in the grip of habit, and to attempt their conversion is a hopeless task. Under all these influences the " stock mixture " and kindred devices continue to flourish, and it is not surprising to learn that the Insurance patients are to come in for their share.

The latest suggestion towards economical prescribing under the Insurance Act is that medicines shall be ordered without the flavouring and sweetening agents which are often added as a tribute to the delicacy of the patient's palate. This, indeed, seems a hard saying, though it might help to modify the enthusiasm of the inveterate medicine-swallower. Admirable, indeed, is any effort towards economy, but what the Insurance Act compels in this direction dims in some degree the bright vision which the patient was to realise under its beneficent sway.

